# STRESS AND RESILIENCE ACROSS PHASES OF COVID-19: A QUALITATIVE STUDY WITH OLDER CHINESE IMMIGRANTS

**DOI:** 10.1093/geroni/igac059.249

**Published:** 2022-12-20

**Authors:** Yi-Hsuan Tung, Kexin Yu, Jiaming Liang, Shinyi Wu, Iris Chi

**Affiliations:** University of Southern California, Los Angeles, California, United States; Oregon Health & Science University, Palo Alto, California, United States; University of Southern California, Los Angeles, California, United States; University of Southern California, Los Angeles, California, United States; University of Southern California, Los Angeles, California, United States

## Abstract

The COVID-19 pandemic has challenged older Chinese immigrants’ lives in physical, psychological, social, and spiritual aspects. This study employed a socioecological perspective of resilience to examine how older Chinese immigrants perceived and navigated through pandemic-related adversities. We conducted a time-bound retrospective qualitative investigation to capture participants’ lived experiences between December 2019 to August 2021. Three phases of the pandemic-related adversities were identified, including uncertain threats and psychological impacts at the beginning, unmet needs and fatigue at 2nd and 3rd wave of infections, and benefit-risk balance after vaccinated. Despite adversities, the integration of strengths, opportunities, and social services at the individual, interpersonal, and neighborhood levels allows participants to appraise and individualize their problem-focus coping (e.g., risk mitigation), selective engagement (e.g., maintaining habits through other means), or emotion-focus coping strategies (e.g., acceptance). Findings highlight the importance of personal and community resources in fostering resilient responses.

